# Imaging characteristics of ovarian sclerosing stromal tumor

**DOI:** 10.1186/s12905-022-01949-x

**Published:** 2022-09-01

**Authors:** Zhifeng Zhou, Xiaoqiao Chen, Zhibin Zeng, Fangjing Zhang, Jing Yan, Gangqiang Hou

**Affiliations:** 1 Radiology Department, Shenzhen Mental Health Center, Shenzhen Kangning Hospital, No.77 Zhenbi Road, Pingshan District, Shenzhen, 518020 Guangdong China; 2 Department of Radiology, Huazhong University of Science and Technology Union Shenzhen Hospital, Shenzhen, 518052 Guangdong China; 3 Affiliated Shenzhen Maternity & Child Healthcare Hospital, Southern Medical University, Shenzhen, 518047 Guangdong China; 4 Department of MRI, The First Affiliated Hospital of Zhengzhou University, Zhengzhou, 450052 Henan China

**Keywords:** Ovary, Sclerosing stromal tumour, Magnetic resonance imaging, Computed tomography

## Abstract

**Objectives:**

This study was designed to evaluate the specific imaging features of ovarian sclerosing stromal tumor (SST), improve its accuracy as well as the specificity of imaging diagnosing, and prevent overestimation of malignancy to reduce unnecessary surgical procedures.

**Methods:**

Eight patients with magnetic resonance imaging (MRI) and six with computed tomography (CT) images were analyzed in this retrospective observational study. All the cases were confirmed by postoperative pathological examination as those of ovarian SST. Imaging and pathological features were also evaluated.

**Results:**

All the 14 masses displayed cystic and solid components with outer surface of tumors contained a capsular and complete smooth rim. Eight tumors of MRI exhibited “lake-island” sign on T2 weighted imaging (T2WI). Two of the 6 CT cases displayed a flaky calcification. One case showed as a multiloculated cystic mass with irregularly thickened septae and the tumor wall. The solid components in other 13 masses were comb- or wheel-like enhanced. After injection of contrast agent, the solid components in 8 cases (57.1%) appeared as early enhancement, whereas the other 6 cases (42.9%) appeared as progressive enhanced, and the cystic components of all the cases had no enhancement in the whole course. Vascular flow signals or/and marked enhancement of the blood vessels were found in 12 lesions (85.7%). Pathological examination demonstrated pseudolobule patterns, round to spindle shaped cells, collagenous areas, edematous hypocellular areas and prominent vasculatures.

**Conclusions:**

The results demonstrated that MRI with “lake-island” signs on T2WI and MRI/CT dynamic enhancement could potentially play a critical role in facilitating appropriate diagnosis preoperatively.

## Introduction

Sclerosing stromal tumor (SST) is a very rare benign sex cord-stromal tumor (SCST) of the ovary, which was initially defined by Chalvardjian and Scully in 1973 [1]. It has been reported that more than 80% of SSTs were observed in young women in the 2nd and 3rd decades of life [2–6]. The most common presenting symptoms include menstrual irregularity, abdominal pain and a lower abdominal mass [2, 7–10], which are not specific.

Histopathologically, SST has a particular pseudolobular architecture, which is primarily composed of the various cellular nodules with hypocellular, edematous and collagenous or fibrous stroma [7, 11, 12]. Upon immunohistochemistry, the expression of different angiogenic factors in SST has been detected, which makes the tumor rich in blood supply [2, 12]. The presence of the solid component and high vascularity upon diagnostic imaging with magnetic resonance imaging (MRI) and/or computed tomography (CT) generally results in this tumor being suspected as the borderline or malignant epithelial tumors and other SCST. Thus, accurate preoperative clinical diagnosis can be relatively difficult. The definite diagnosis of SST is made based on the post-operative pathological examination after oophorectomy, and laparoscopic surgery is seldom performed in affected SST patients. Therefore, accurate preoperative imaging diagnosis for SST can be helpful to prevent overestimation of malignancy, perform minimally invasive surgery and significantly reduce unnecessary surgical procedures, especially among the young women and children [10]. In 2020, the American College of Radiology released the ovarian-adnexal reporting and data system magnetic resonance imaging (O-RADS MRI) risk stratification and management system, with the main goals of optimizing the quality and consistency of imaging reports and avoiding unnecessary surgery in the patients at low risk of malignancy [13, 14].

The present study reviewed MRI/CT features of 14 cases of histologically confirmed ovarian SST and analyzed the possible correlation between their imaging and pathological characteristics. The objective of this study was to primarily evaluate the specific imaging features of ovarian SST, improve its accuracy and specificity of imaging diagnosing, distinguish it from the malignant tumors to avoid unnecessary surgery, and assess the feasibility in the diagnosis of SST classified according to the O-RADS MRI score.

## Materials and methods

### Patients

There were 14 cases (from 2005 to 2018) included in this retrospective study. The MRI/CT images of all the cases were retrospectively reviewed by two experienced radiologists (G.H and X.C) who were blinded to the pathological findings of these selected cases. When the diagnosis was inconsistent, a consensus was reached through discussion. The clinical details that were reviewed included age at the time of MRI/CT scanning, clinical presentation, location and size of the tumors, pre-op imaging diagnosis, and O-RADS MRI score (Table 1). The study protocol was approved by the Shenzhen Kangning Hospital Institutional Review Board (Approval code: 2022-05-16-2).

**Table 1:** Clinical findings in cases of ovarian sclerosing stromal tumor

Case No	Age (years)	Clinical presentation	Side/size of tumor (cm)	Pre-op imaging diagnosis	O-RADS MRI score
MR01	27	Adnexal mass	LO/6.2 × 5.8	Malignant ovarian tumors	4
MR02	33	Pelvis mass	LO/8 × 7	Ovarian SST	5
MR03	15	Pelvis mass	RO/8 × 6	Ovarian SST	4
MR04	22	Pelvis mass	LO/8 × 6	Ovarian SST	5
MR05	24	Irregular menstruation	RO/7.2 × 5.6	Ovarian SST	5
MR06	14	Pelvis mass	RO/7.8 × 5.2	Ovarian hypervascular tumor	5
MR07	26	Adnexal cystic mass	LO/16.8 × 14.7	Ovarian myxoma	3
MR08	27	Adnexal mass, pelvic pain	RO/4.2 × 3.6	Ovarian hypervascular tumor	5
CT01	24	Adnexal mass	RO/8.6 × 5.9	Malignant adnexal tumors	–
CT02	27	Normal	RO/2.5 × 1.8	Malignant adnexal tumors	–
CT03	12	Pelvis mass	LO/11.6 × 10	Pelvic cystic-solid mass	–
CT04	18	Pelvis mass	LO/8.1 × 7.9	Ovarian SST	–
CT05	47	Pelvis mass	RO/10.3 × 9.3	Pelvic cystic-solid mass	–
CT06	22	Irregular menstruation	LO/6.6 × 4.9	Ovarian SST	–

*RO* right ovary, *LO* left ovary

### Image capture and evaluation

Eight cases of MRI examinations were performed on 1.5T or 3.0T MR scanners. The sequences included T1 weighted imaging (T1WI), T2 weighted imaging (T2WI), and the dynamic enhanced scanning after intravenous injection of gadolinium-containing contrast agent. Six cases of CT examinations were performed with plain scan and the dynamic enhanced scanning after intravenous injection of iodine-containing contrast agent. The imaging characteristics that were collected included location, main component, calcification (CT cases), capsular rim, “Lake-island” sign, enhancement patterns, vascular flow/marked enhancement, comb-shaped wall nodules after enhancement, and without the presence of the swollen lymph nodes around the mass.

### Pathological presentations

The pathological characteristics that were reported included the gross appearance, tumor size, alternating pattern and cell type of hypercellular and hypocellular areas, hemangiopericytoma-like vascular pattern and histopathological abnormalities of the stroma, such as edema, cystic degeneration, as well as the presence of myxoid or necrotic tissues.

## Results

Fourteen patients enrolled in this study ranged in age from 12 to 47 years (Table 1). The mean age was 24.1 ± 8.8 years. The main clinical presentation was an adnexal or pelvis mass, 2 patients had irregular menstruation and 1 patient was normal, but only 1 case complained about the pelvic pain. Among the 14 cases with SST, 7 of them were located on the left ovary and rest 7 cases on the right. The size of the diagnosed tumors varied from 2.5 to 16.8 cm in the largest diameter (Table 2).

**Table 2:** Imaging characteristics data of ovarian sclerosing stromal tumor

Imaging features	The number of cases	Mean age of patient (range, years)	Mean size of tumour (range, cm)
Location (L/R)	7/7	22.9 (12–33)/25.4 (15–47)	9.3 (6.2–16.8)/7.0 (2.5–10.3)
Main component (solid/cystic)	13/1	24 (12–47)/26	8 (2.5–11.6)/16.8
Calcification on CT	2	21 (18–24)	8.4 (8.1–8.6)
Capsular rim	14	24.1 (12–47)	8.1 (2.5–16.8)
“Lake-island” on T2WI	8	23.5 (14–33)	8.3 (4.2–16.8)
Progressive continual enhancement	6	23.8 (12–33)	7.2 (2.5–8)
Early-fast sustained enhancement	8	24.4 (14–47)	8.9 (4.2–16.8)
Vascular flow/marked enhancement	12	24.7 (12–47)	7.9 (4.2–11.6)
Comb-shaped wall nodule	13	24 (12–47)	7.5 (2.5–11.6)
Swollen lymph nodes	0	0	0

The mean size of the tumor was calculated by the largest diameter of the tumor

**Fig. 1:**
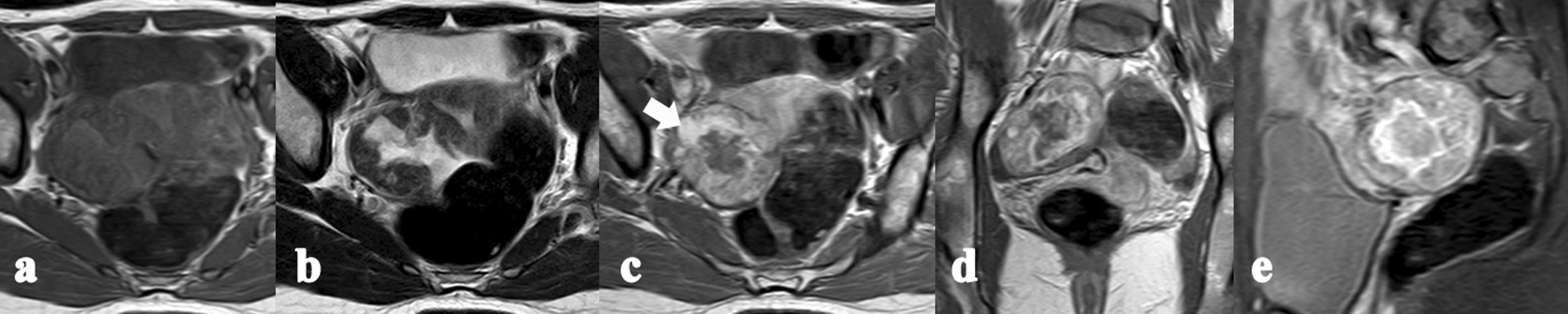
Case MR03. Axial T1WI (**a**) and T2WI (**b**) showing a round and well-defined heterogenous mass with solid and cystic components; **c**–**e** dynamic enhancement images showing early peripheral ring enhancement in the axial arterial phase (**c**), and associated with progressive and prolonged enhancement in the inner zone of the lesion in the coronal venous phase (**d**) and sagittal delayed phase (**e**). Part of the hyperintensity area on T2WI of the mass showed enhancement (**c**, white arrow)

**Fig. 2:**
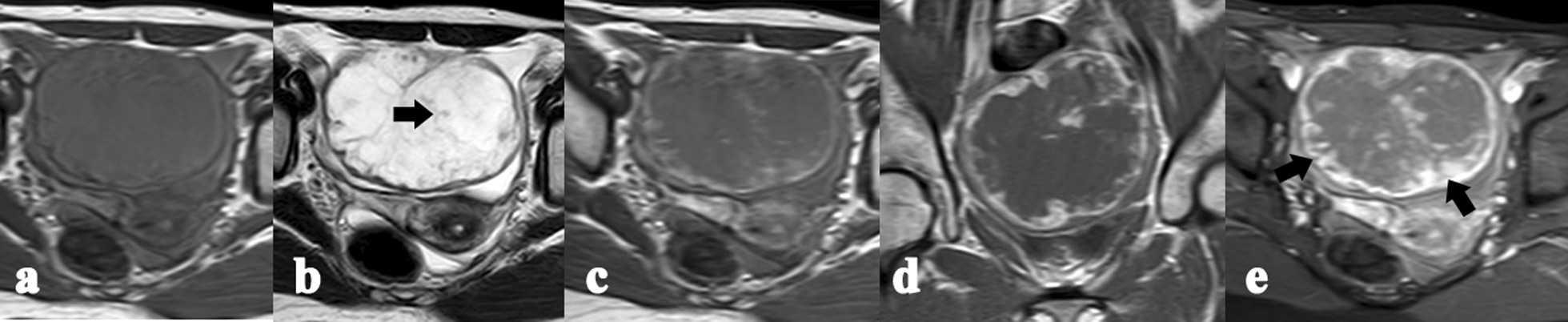
Case MR04. Axial T1WI (**a**) depicts a welldemarcated pelvic mass with low signal intensities. Axial T2WI (**b**) shows the mass with heterogeneous high signal intensity and a peripheral low signal rim. The scattered clusters and linear iso-hypointensity areas at the hyperintensity background within the neoplasm, i.e., “lake-island” sign, on T2WI (**b**, black arrow). Dynamic enhancement images showing early peripheral ring enhancement (**c**), and progressive and prolonged enhancement in the inner zone of the lesion in the late phases (**d**, **e**). The solid components in mass are comb- or wheel-like enhanced (**e**, black arrows)

**Fig. 3:**
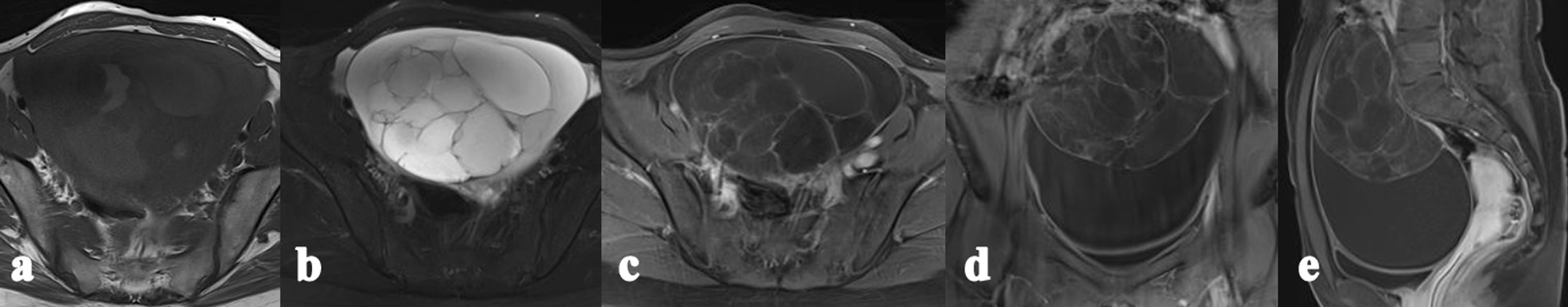
Case MR07. Axial T1WI (**a**) and T2WI (**b**) showing a multiloculated cystic mass with irregularly thickened septae and tumor wall; **c**–**e** dynamic enhancement images showing early-fast sustained enhancement

**Fig. 4:**
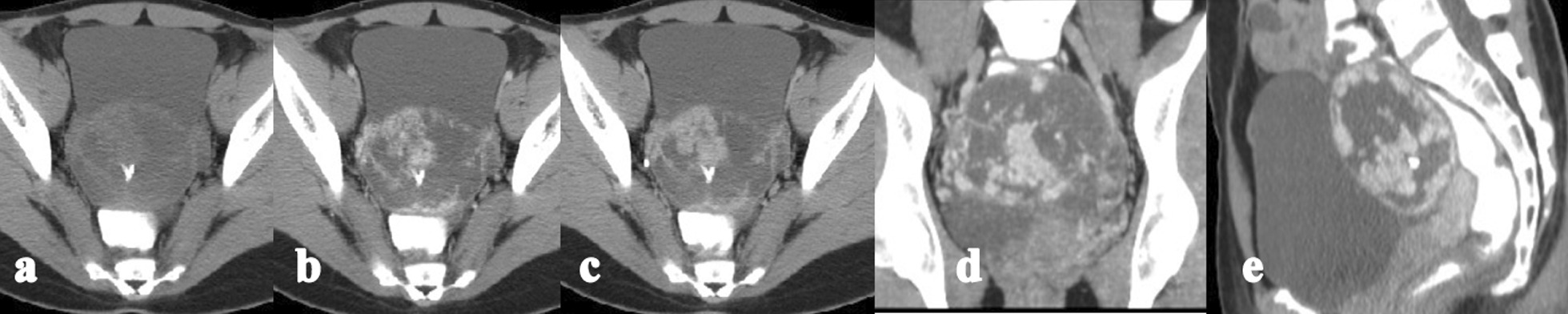
Case CT01. Axial plain CT scan (**a**) showing a round and well-defined heterogenous mass, and a patchy calcification in the center of the mass; **b**–**e** dynamic enhancement CT scan showing early-fast sustained enhancement

Radiologically, plain CT scans showed mixed and low density, whereas T1WI displayed hypointensity, but mixed and hyperintensity on T2WI. Thirteen diagnosed tumor masses (92.9%) showed heterogeneously solid and cystic components, and 1 case (7.1%) was predominantly cystic tumor. Two of the 6 CT cases (1/3) exhibited a patchy calcification. In all the cases, the outer surface of the tumor contained a capsular and complete smooth rim. Twelve cases of MRI/CT images displayed little ascites around the mass (85.7%). However, two cases showed no ascites, but a small amount of light ascites were observed during the surgery. All of the 8 MRI cases (100%) displayed scattered iso-hypointensity areas at the hyperintensity background within the neoplasm, i.e., “lake-island” sign, on T2WI. According to the dynamic enhancement pattern, the solid components in 8 tumors (57.1%) appeared early-fast sustained enhancement, but the solid components of the other 6 cases (42.9%) showed progressive centripetal continual enhancement, whereas the cystic components of all the cases exhibited no substantial enhancement in the whole course. During the delayed phases, the solid components in 13 masses were comb- or wheel-like enhanced, but the case MR07 presented as a multiloculated cystic mass with irregularly thickened septae and the tumor wall. Moreover, vascular flow signals and/or marked enhancement of the blood vessels were observed in 12 lesions (Table 2, Figs. 1, 2, 3, 4). In addition, no pelvic or lumbo-aortic enlarged lymph nodes were observed. Furthermore, during the preoperative imaging diagnosis, 6 cases (42.9%) were confirmed, 4 cases (28.6%) were misdiagnosed, and 4 cases (28.6%) were not clearly diagnosed. In all MRI cases, 1, 2, and 5 cases with O-RADS MRI scores of 3, 4, and 5, respectively were observed (Table 1).

**Fig. 5:**
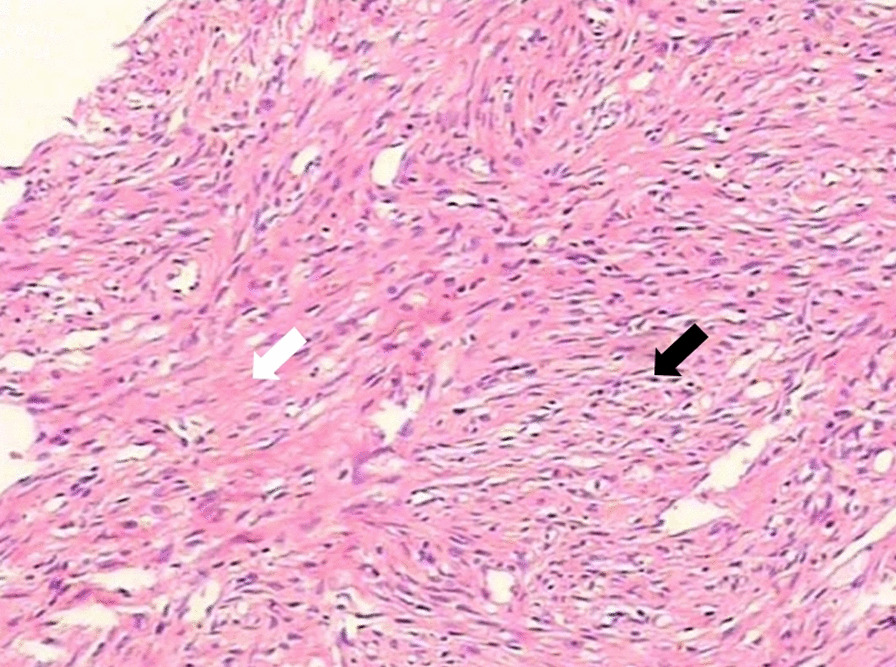
Microscopic image reveals a pseudolobular appearance composed of admixture of cellular nodules (black arrow) separated by collagenous, edematous hypocellular areas (white arrow) (H&E staining, 200)

In addition, upon then microscopic examination, the appearance of tumor was found to be significantly variable. All the tumors were well circumscribed. Thirteen neoplasms were solid and cystic, whereas the one other was predominantly cystic. Histologically, all the cases demonstrated pseudolobular appearance of the cellular areas, collagenous and edematous hypocellular areas. And in the cellular areas, round or oval-shaped and spindle cells were predominantly present (Fig. 5). Twelve out of 14 cases presented hemangiopericytoma-like vasculatures.

## Discussion and conclusions

Ovarian SST is a considerably rare benign SCST that occurs commonly in the young women during childbearing age. According to the previous reports, the patients’ ages ranged from 5 to 80 years (mean age 28 years) [1, 7, 11, 15]. In our 14 cases, patients belonged to the younger subgroup, ranging in age from 12 to 47 years (mean age 24.1 years). SST has variable and non-specific clinical presentations, which usually include irregular menstruation that was reported to disappear after surgical excision of the tumor [16, 17], adnexal mass and/or pelvic pain. Additionally, the tumors might in some rare cases, cause masculinization, anovulation, infertility and Meigs’ syndrome [16, 18–20].

Histopathologically, SST is characterized by a cell-rich pseudolobular architecture in edematous or fibrous stroma [7, 11]. The cell-rich areas consist primarily of spindle, round or oval-shape, and polygonal cells. The tumors also exhibit hemangiopericytoma-like vascular pattern and express different angiogenic factors [11, 21]. In addition, the tumors occasionally present the patchy calcification in the hypocellular areas [11, 20], which are in line with the case of CT01 and CT04 in this study.

Pathology and imaging findings can be used for mutual confirmation, i.e., imaging findings can reflect pathological tissue structure and cellular distribution, and pathological changes can explain the various imaging findings at the molecular level. A number of the previous studies have demonstrated that the density or signal and enhancement patterns of SST were associated with cellularity, vascularity, collagenous distribution and cystic or necrosis architecture observed during the histopathological examination of the tumor [6, 22]. In the present study, we found that most of the identified SSTs were accompanied by “lake-island” sign on T2WI and progressive centripetal/early-fast continual and comb- or wheel-like enhancement. These imaging findings reflect the characteristic microscopic pattern of the tumor in which the pseudo-separation of cell-rich region is mainly separated by hypocellular region of loose collagen and edematous connective tissue. In T2WI “lake-island” sign, combined with T1WI images, the scattered clusters and linear isointensity and/or hypointensity area on T2WI, as well as isointense to normal muscle on T1WI, indicated the corresponding area of cell-rich residual parenchymal, means “island”. On the contrary, the hyperintensity area on T2WI, but hypointensity on T1WI, represented the cystic degeneration area, means “lake”. The SST is the only subtype of benign SCST that, when enhanced distinctly, can predict the hypervascularity of the tumor [9]. Pathologically, prominent vascular and collagen components can explain the early and continual obviously enhancement. For example, in the artery phase after administration of intravenous contrast material, early and distinctive enhancement of the rim of the tumor was possible due to the hypervascularity in this space. However, in the delayed phase, the area of the prolonged and progressive enhancement observed in the inner zone of mass was considered to be associated with the collagenized hypocellular area, which showed hyperintensity on T2WI [6]. The present imaging findings were in line with the previous studies [6, 8, 22–24]. Thus, the occurrence of early or prolonged enhancing was dependent primarily on whether the vascular component or collagen component was dominant in the tumor. In our preoperative imaging diagnosis, there were 3 cases misdiagnosed as the malignant tumors, and 2 cases were diagnosed as ovarian hypervascular mass, which might be due to less SST cases reported in the prior literature and insufficient clinical experience. In addition, 2 cases were diagnosed as pelvic cystic-solid mass because the mass was too large to locate and characterize accurately. Besides, one single case (case MR07) was diagnosed with ovarian myxoma, and classified as low risk lesion with O-RADS MRI score 3, due to the multiocular cystic imaging features, but postoperative pathology confirmed SST of ovary. Roth and colleagues [11] have previously reported 3 SSTs undergoing a transition to ovarian myxoma, which were in line with our case. This unusual histologic transformation could make the diagnosis more challenging and requires careful identification.

Due to the solid components of SST enhanced vividly, 7 of 8 MRI cases had O-RADS MRI score of 4–5, which were classified as intermediate-high-risk lesions. This result contradicted the fact that SST is a benign tumor. However, O-RADS MRI risk score governing concepts have proposed clearly that some characteristic lesions can be confidently diagnosed on MRI regardless of the O-RADS MRI score category. In addition, previous studies have also considered that some benign masses, such as fibroadenoma, may be treated as high-risk lesions in the practical settings by using O-RADS MRI score [25]. Collectively, the characteristic imaging manifestations of SST can play an important role in preoperative diagnosis.

The differential diagnoses of SSTs include other benign SCSTs, such as fibromas or thecomas, metastatic and malignant epithelial ovarian tumors [26]. Most benign SCSTs are cytologically denser and more homogeneous as compared to SST, similar to their differences in imaging. In addition, SST contains characteristic vasculature and is markedly enhanced, which is not found in the fibromas or thecomas. However, clear difference from malignant ovarian cancer might not always simple, given that the solid components of the tumor can enhance vividly for the dysregulation in vascular endothelial growth factor production. Nevertheless, no pelvic and para-aortic lymph nodes were swollen, which was an indirect indication suggesting that the tumor was benign. Therefore, “Lake-island” signs on T2WI, comb- or wheel-like nodules with strong enhancement a peripheral hypointense rim with no swollen lymph nodes were the main findings of SST. In addition, no signs of local or distal recurrences have been reported in our patients until now.

In summary, we have described and analyzed the MRI/CT findings and pathological features in ovarian SST, which can be useful to improve the preoperative diagnosis of the tumor. Dynamic enhancement scanning plays a critical role in the diagnosis of the tumor by revealing the typical contrast enhancement pattern of SST. T1WI combined with T2WI and the size of pelvic or lumbo-aortic lymph nodes might be used as complementary imaging information to show the stromal component and identify the benign or malignant characteristic of the tumor, respectively. Overall, combining all imaging findings can aid the radiologists to make a specific diagnosis of SST, which may lead to a less-invasive and unnecessary surgical procedure in young women and girls. Our findings clearly identify distinctive imaging features associated with SST and expand our knowledge about their imageology.

## Data Availability

The datasets used and analysed during the current study are not publicly available due to the privacy protection of the subjects, but are available from the corresponding author on reasonable request.

## Abbreviations

SST: Ovarian sclerosing stromal tumor
SCST: Sex cord-stromal tumor
MRI: Magnetic resonance imaging
CT: Computed tomography
O-RADS: Ovarian-adnexal reporting and data system
T1WI: T1 weighted imaging
T2WI: T2 weighted imaging
